# Maternal stress induced endoplasmic reticulum stress and impaired pancreatic islets’ insulin secretion via glucocorticoid receptor upregulation in adult male rat offspring

**DOI:** 10.1038/s41598-022-16621-5

**Published:** 2022-07-22

**Authors:** Mina Salimi, Farzaneh Eskandari, Fateme Binayi, Afsaneh Eliassi, Hossein Ghanbarian, Mehdi Hedayati, Javad Fahanik-babaei, Mohamad Eftekhary, ‬Rana Keyhanmanesh, Homeira Zardooz

**Affiliations:** 1 Department of Physiology, Faculty of Medicine, Tabriz University of Medical Sciences, PO Box: 5166614756, Tabriz, Iran; 2 Department of Physiology, School of Medicine, Shahid Beheshti University of Medical Sciences, PO Box: 19615-1178, Tehran, Iran; 3 Neurophysiology Research Center, Shahid Beheshti University of Medical Sciences, Tehran, Iran; 4 Cellular and Molecular Biology Research Center, Shahid Beheshti University of Medical Sciences, Tehran, Iran; 5 Cellular and Molecular Endocrine Research Center, Research Institute for Endocrine Sciences, Shahid Beheshti University of Medical Sciences, Tehran, Iran; 6 Electrophysiology Research Center, Neuroscience Institute, Tehran University of Medical Sciences, Tehran, Iran; 7 Drug Applied Research Center, Tabriz University of Medical Sciences, Tabriz, Iran

**Keywords:** Physiology, Metabolism

## Abstract

Exposure to perinatal (prenatal and/or postnatal) stress is considered as a risk factor for metabolic disorders in later life. Accordingly, this study aimed to investigate the perinatal stress effects on the pancreatic endoplasmic reticulum (ER) stress induction, insulin secretion impairment and WFS1 (wolframin ER transmembrane Glycoprotein, which is involved in ER homeostasis and insulin secretion) expression changes, in rat offspring. According to the dams’ period of exposure to variable stress, their male offspring were divided into, control (CTRL); pre-pregnancy, pregnancy, lactation stress (PPPLS); pre-pregnancy stress (PPS); pregnancy stress (PS); lactation stress (LS); pre-pregnancy, pregnancy stress (PPPS); pregnancy, lactation stress (PLS); pre-pregnancy, lactation stress (PPLS) groups. Offspring pancreases were removed for ER extraction and the assessment of ER stress biomarkers, WFS1 gene DNA methylation, and isolated islets’ insulin secretion. Glucose tolerance was also tested. In the stressed groups, maternal stress significantly increased plasma corticosterone levels. In PPS, PS, and PPPS groups, maternal stress increased Bip (Hsp70; heat shock protein family A member 4), Chop (Ddit3; DNA- damage inducible transcript3), and WFS1 protein levels in pancreatic extracted ER. Moreover, the islets’ insulin secretion and content along with glucose tolerance were impaired in these groups. In PPS, PS, LS and PPPS groups, the pancreatic glucocorticoid receptor (GR) expression increased. Maternal stress did not affect pancreatic WFS1 DNA methylation. Thus, maternal stress, during prenatal period, impaired the islets’ insulin secretion and glucose homeostasis in adult male offspring, possibly through the induction of ER stress and GR expression in the pancreas, in this regard the role of WFS1 protein alteration in pancreatic ER should also be considered.

## Introduction

Impaired insulin secretion from the islets of Langerhans is one of the major causes of metabolic disorders associated with glucose metabolism such as diabetes, which could be caused by several environmental factors including stress^1^. Prevalence of metabolic disorders was thought to be mainly affected by genetic factors; however, genetics cannot explain all reasons causing metabolic disorders, thus recent research has focused on lifestyle as a major factor for the incidence of metabolic diseases^2^. Maternal stress/perinatal stress is considered to be a risk factor in terms of trans-generational consequences. It refers to maternal exposure to stressful conditions such as psychological and physical distress (e.g., depression, anxiety, high carbohydrate and/or high fat diets) during preconception, pregnancy and also lactation periods, which can affect the programming of developing organs of offspring, and may lead to negative outcomes, such as cardiovascular dysfunction and diabetes in later life^3,4^. The critical mechanisms proposed to explain these associations are poorly understood. Epigenetic changes in various body systems, induced by elevated levels of glucocorticoids, has been suggested as a potential mechanism linking adverse experiences during perinatal period and impaired offspring health in later life^5^. Many human and animal studies have verified that perinatal exposure to high levels of glucocorticoids can, directly and indirectly, change the expression levels of ‘epigenetic regulator’ genes such as DNA methyltransferase 1 (Dnmt1) in offspring, through free fatty acids, inflammatory cytokines, oxidative stress, and hyperglycemia^6^. Alteration of offspring hypothalamus–pituitary–adrenal (HPA) axis programming (and hence circulating glucocorticoid levels) may result in an increased HPA responsiveness to stress in adulthood^7^**.**

Recent studies have demonstrated that DNA methylation was sensitive to early life exposure to environmental stress. As proof, adult offspring with a history of child abuse and neglect showed anxiety and HPA axis dysfunction in adulthood and these offspring exhibited hyper-methylated and reduced expression of hippocampal glucocorticoid receptors (GR)^8^. In contrast, higher early postnatal maternal care such as lick-groom and arched-back nursing (LG-ABN) behaviors towards the pups, caused hypo-methylation and elevated GR expression in the hippocampus and reduced responses to stress^9^. Furthermore**,** Nyirenda et al. indicated that exposure to excessive glucocorticoids during pregnancy and lactation had long-lasting effects on the programming of metabolic signaling pathways which manifested as hyperglycemia, glucose intolerance, and insulin resistance in adult animals^10^. On the other hand, it has recently been shown that the stress of endoplasmic reticulum (ER) is a central mechanism involved in the onset of diseases related to metabolic disorders. Endoplasmic reticulum (ER) homeostasis is crucial for the appropriate function of cells, and a safeguard system called unfolded protein response (UPR) is activated consequent to impaired ER homeostasis. This UPR seeks to alleviate the adverse effects of ER stress by activating three signaling pathways: IRE1, PERK and ATF6, which are referred to as UPR sensors^11,12^.

A study has demonstrated that environmental and genetic factors were the causes of ER stress induction, specifically in β cells, which may lead to the reduction of insulin synthesis and secretion^13^. Moreover, Linssen et al. reported that prednisolone treatment could induce the GR-mediated activation of IRE1-XBP1 pathways that resulted in the suppression of insulin biosynthesis and secretion in the insulin-secreting INS-1E cells^14^. Other studies have also shown that low-dose of corticosterone could induce ER stress and elevate markers of ER stress (Bip, Chop) via the GR in macrophages^15^ and pancreas^16^ of C57BL/6 J mice. Furthermore, it has revealed that WFS1 played an important role in insulin biosynthesis and secretion as well as maintaining ER homeostasis in pancreatic β-cells^17^. WFS1 is a trans-membrane protein located in the ER and it is a novel component of ER stress signaling^18–20^. Under ER stress conditions, expression of the WFS1 gene was elevated via the X-box binding protein (XBP1), and mitigates ER stress response in the pancreatic β-cells^19^. Loss of its function caused impairment in ER stress response and apoptosis^18,21^. A number of recent studies have reported that mutations in this protein increased plasma glucose and reduced plasma insulin^22^, impaired glucose-stimulated insulin secretion and insulin content of the islets of Langerhans^21^, decreased beta cell mass, and elevated the expression of ER stress markers^23^. Given the effects of stress exposure and consequent corticosterone elevation on the induction of ER stress, and the important role of WFS1 protein in modulating the UPR pathway and β-cell insulin biosynthesis and secretion, this investigation was proposed to test the hypothesis that exposure to maternal stress during pre-pregnancy, pregnancy and lactation periods would affect programming of the systems involved in glucose homeostasis in adult male rat offspring through high corticosterone levels, induction of pancreatic ER stress and/or changes in pancreatic WFS1 gene expression and DNA methylation.

## Results

### Maternal plasma corticosterone levels

**Figure 1:**
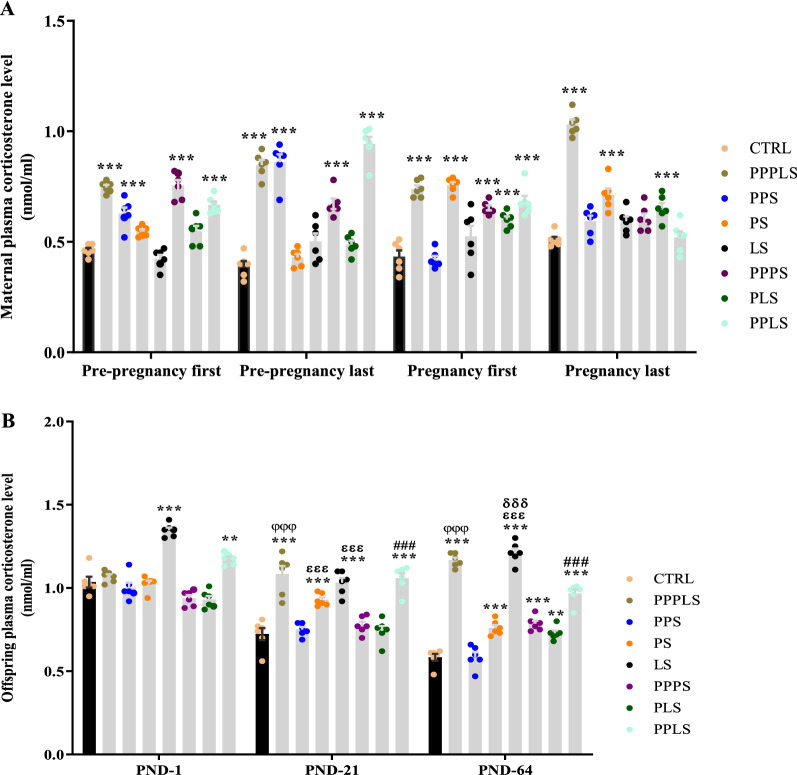
Effect of perinatal stress on corticosterone levels of maternal (**A**) and male offspring (**B**). Each column represents mean ± SEM (6 rats/group, 6 litters/group). Data was analyzed using two-way repeated measures ANOVA followed by Tukey’s post hoc test. The analysis were performed in each separate category; ^**^P < 0.01, ^***^P < 0.001 versus CTRL group; ^###^P < 0.001 versus PPS group; ^φφφ^P < 0.001 versus PPPS group; ^ɛɛɛ^P < 0.001 versus PLS group; ^δδδ^P < 0.001 versus PPLS group. *CTRL* non stress, *PPPLS* pre-pregnancy, pregnancy, lactation stress, *PPS* pre-pregnancy stress, *PS* pregnancy stress, *LS* lactation stress, *PPPS* pre-pregnancy, pregnancy stress, *PLS* pregnancy, lactation stress, *PPLS* pre-pregnancy, lactation stress.

The two-way repeated measures ANOVA showed that during pre-pregnancy period, after the first and the last exposure to stress, maternal variable stresses significantly increased plasma corticosterone levels in PPPLS, PPS, PPPS, and PPLS groups compared to CTRL group (P < 0.001). During pregnancy period, after the first exposure to stress, maternal plasma corticosterone levels in PPPLS, PS, PPPS, PLS, and PPLS groups showed marked elevations (P < 0.001) and also, after the last exposure to stress, in PPPLS, PS, and PLS groups (P < 0.001) compared to CTRL group (Fig. 1A).

### Plasma corticosterone levels in offspring

The two-way repeated measures ANOVA showed that at PND-1, there was a significant increase in the plasma corticosterone levels of offspring in LS (P < 0.001) and PPLS (P < 0.01) groups compared to CTRL group, however; no changes were observed in the offspring plasma corticosterone levels of other study groups compared to CTRL group. At PND-21, the plasma corticosterone levels of offspring showed significant elevation in PPPLS, PS, LS and PPLS groups compared to CTRL group (P < 0.001). In addition, at PND-64, plasma corticosterone levels of offspring in PPPLS, PS, LS, PPPS, PPLS (P < 0.001) and PLS (P < 0.01) groups displayed significant increment compared to CTRL group (Fig. 1B).

### Plasma glucose and insulin levels during OGTT

**Figure 2:**
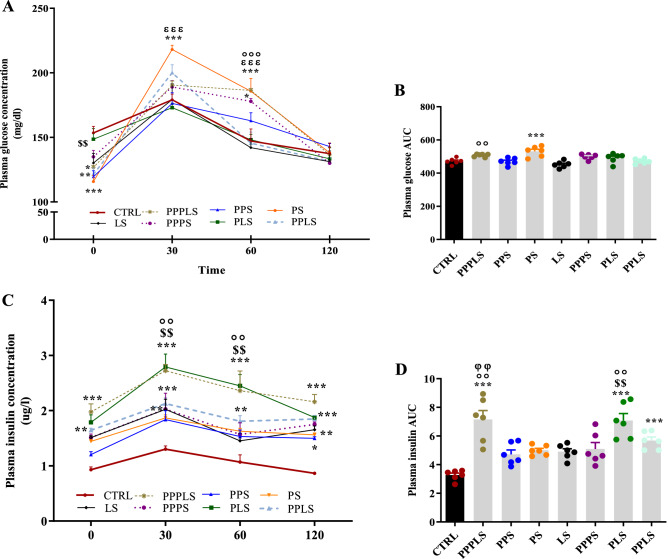
Effect of perinatal stress on plasma glucose level (**A**) and AUC (**B**), plasma insulin level (**C**) and AUC (**D**) during OGTT in young adult male offspring. Each point and column represent mean ± SEM (n = 6 rats/group, 6 litters/group); Data was analyzed using two-way repeated measures ANOVA followed by Tukey’s post hoc test; ^*^P < 0.05, ^**^P < 0.01, ^***^P < 0.001 versus CTRL group; ^$$^P < 0.01 versus PS group; ^οο^P < 0.01, ^οοο^P < 0.001 versus LS group; ^φφ^P < 0.01 versus PPPS group; ^ɛɛɛ^P < 0.001 versus PLS group. *CTRL* non stress, *PPPLS* pre-pregnancy, pregnancy, lactation stress, *PPS* pre-pregnancy stress, *PS* pregnancy stress, *LS* lactation stress, *PPPS* pre-pregnancy, pregnancy stress, *PLS* pregnancy, lactation stress, *PPLS* pre-pregnancy, lactation stress.

Plasma glucose levels showed significant decrease in PPPLS (P < 0.05), PPS and PPLS (P < 0.01) PS (P < 0.001) groups compared to CTRL group in fasting conditions (0 min). After the glucose load, the plasma levels of glucose elevated at time 30, and these levels in the PS group were significantly higher than that of CTRL group (P < 0.001), then the values began to drop in all study groups; however, plasma glucose levels at 60 min were significantly higher in PPPLS, PS (P < 0.001) and PPPS (P < 0.05) groups than those of CTRL group (Fig. 2A). The area under the curve (AUC) of plasma glucose levels in PS group showed that plasma glucose levels increased significantly compared to CTRL group. These data indicate that glucose clearance in PS group was significantly lower compared to CTRL group (P < 0.001, Fig. 2B).

Plasma insulin levels significantly increased in PPPLS, PLS (P < 0.001) and PPLS (P < 0.01) groups compared to CTRL group in fasting condition (0 min). The values elevated and reached a peak after 30 min of glucose load and approached those of time zero within 120 min. Plasma insulin levels at min 30 in all stress groups except PS and PPS group were higher than that of CTRL group (P < 0.001 to P < 0.01). This parameter at min 60 in PPPLS, PLS (P < 0.001) and PPLS (P < 0.01) groups was higher than that of CTRL group. Plasma insulin levels at min 120 in all stress groups displayed marked increment compared to CTRL group (P < 0.001 to P < 0.05, Fig. 2C). The area under the curve (AUC) of plasma insulin levels in PPPLS, PLS and PPLS groups showed that plasma insulin levels increased significantly compared to CTRL group. These results suggest that these groups may be more efficient in clearing the glucose load (P < 0.001, Fig. 2D).

### Indices of insulin resistance (HOMA-IR) and insulin sensitivity (Matsuda index)

**Figure 3:**
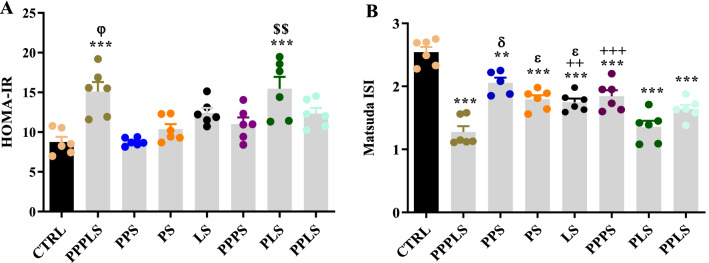
Effect of perinatal stress on HOMA-IR (**A**) and Matsuda index (**B**) in young adult male offspring. Each column represents mean ± SEM (6 rats/group, 6 litters/group); Data was analyzed using one-way ANOVA followed by Tukey’s post hoc test; ^**^P < 0.01, ^***^P < 0.001 versus CTRL group; ^++^P < 0.01, ^+++^P < 0.001 versus PPPLS group; ^**$$**^P < 0.01 versus PS group; ^φ^P < 0.05 versus PPPS group; ^ɛ^P < 0.05 versus PLS group; ^δ^P < 0.05 versus PPLS group. *CTRL* non stress, *PPPLS* pre-pregnancy, pregnancy, lactation stress, *PPS* pre-pregnancy stress, *PS* pregnancy stress, *LS* lactation stress, *PPPS* pre-pregnancy, pregnancy stress, *PLS* pregnancy, lactation stress, *PPLS* pre-pregnancy, lactation stress.

As shown in Fig. 3A, the HOMA-IR index was elevated markedly in the PPPLS and PLS groups compared to CTRL group (P < 0.001). There were no significant differences in relation to this index in other stress groups compared to CTRL group, however; HOMA-IR index in PPPLS group increased significantly compared to PPPS groups (P < 0.05). Also, this index in the PLS group elevated significantly compared to PS group (P < 0.01, Fig. 3A).

A significant decrease in Matsuda index was observed in all stress groups compared to CTRL group (P < 0.001 to P < 0.01). Also, there was a significant reduction of this index in PPPLS group compared to PPPS and LS groups (P < 0.001 to P < 0.01). On the other hand, Matsuda index in PPS, PS and LS groups was significantly higher than that of PPL and PLS groups (P < 0.05, Fig. 3B).

### Glucose-stimulated insulin secretion

**Figure 4:**
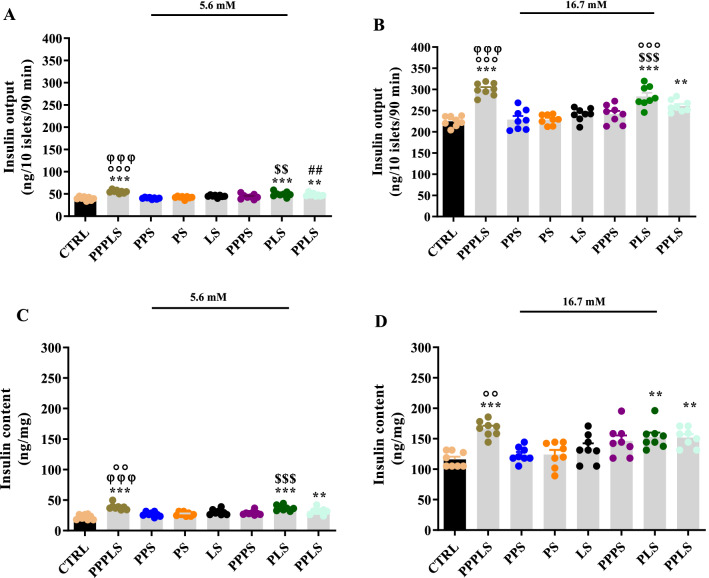
Effect of perinatal stress on the isolated islets insulin secretion (**A**,**B**) and insulin content (**C**,**D**) in response to 5.6 mM and 16.7 mM glucose concentrations in the young adult male offspring. Each column represents mean ± SEM (4 rats/group, 4 litters/group). Data was analyzed using one-way ANOVA followed by Tukey’s post hoc test; ^**^P < 0.01, ^***^P < 0.001 versus CTRL group; ^##^P < 0.01 versus PPS group; ^$$^P < 0.01, ^$$$^P < 0.001 versus PS group; ^οο^P < 0.01, ^οοο^P < 0.001 versus LS group; ^φφφ^P < 0.001 versus PPPS group. *CTRL* non stress, *PPPLS* pre-pregnancy, pregnancy, lactation stress, *PPS* pre-pregnancy stress, *PS* pregnancy stress, *LS* lactation stress, *PPPS* pre-pregnancy, pregnancy stress, *PLS* pregnancy, lactation stress, *PPLS* pre-pregnancy, lactation stress.

In all study groups, the insulin secretion from isolated islets in the presence of 16.7 mM glucose were higher than 5.6 mM glucose concentration. The basal insulin secretion after incubation in 5.6 mM glucose in PPPLS, PLS and PPLS groups were significantly higher than that of CTRL group (P < 0.001 to P < 0.01). Furthermore, the insulin secretion in the PPPLS group in the presence of 5.6 mM glucose concentration was higher than those of LS, and PPPS groups (P < 0.001). The responses to 5.6 mM glucose in PLS and PPLS groups were also significantly higher than those of PS and PPS groups respectively (P < 0.01, Fig. 4A).

When islets were stimulated with 16.7 mM glucose concentration, the significant increase in insulin secretion was observed in PPPLS, PLS, and PPLS groups compared to the CTRL group (P < 0.001 to P < 0.01). There was the significant increase of insulin secretion in PPPLS group in the presence of 16.7 mM glucose concentration compared to LS, and PPPS groups (P < 0.001). Moreover, the insulin secretion in the presence of 16.7 mM glucose concentration in PLS group was significantly higher than those of PS and LS groups (P < 0.001, Fig. 4B).

### Insulin content in isolated islets

The isolated islets’ insulin content in response to 5.6 mM glucose concentration showed the significant increases in PPPLS, PLS and PPLS groups compared to CTRL group (P < 0.001 to P < 0.01). The significant elevation of the insulin content of islets in PPPLS group in the presence of 5.6 mM glucose concentration was observed compared to PPPS, and LS groups (P < 0.001 to P < 0.01). Moreover, there was a significant increase of islets’ insulin content in PLS group in the presence of 5.6 mM glucose concentration compared to PS group (P < 0.001, Fig. 4C).

Moreover, after stimulation with 16.7 mM glucose, there was a significant increase in the insulin content of isolated islets in PPPLS, PLS and PPLS groups compared to CTRL group (P < 0.001 to P < 0.01). However, this parameter in the presence of 16.7 mM glucose concentration in PPPLS group was significantly higher than those of LS groups (P < 0.01, Fig. 4D).

### Bip mRNA and protein expression levels

**Figure 5:**
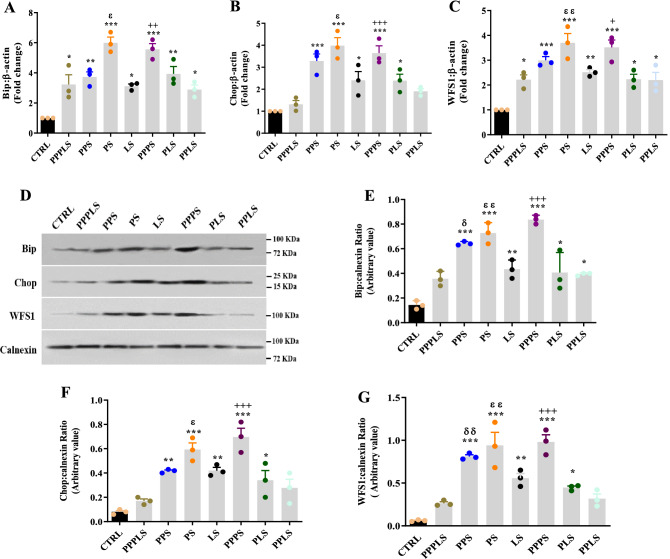
Effect of perinatal stress on pancreatic tissue Bip (**A**), Chop (**B**), and WFS1 (**C**) mRNA expression and pancreatic extracted ER levels of Bip (**D**,**E**), Chop (**D**,**F**) and WFS1 (**D**,**G**) proteins in young adult male offspring. Representative figures for the RT-PCR are shown the fold change (3 rats/group, 3 litters/group) and representative bands are shown the respective densitometric values (8 rats/ group, 8 litters/group). Twenty μg proteins were separated on SDS-PAGE, western blotted, probed with anti-Bip antibody, anti-Chop antibody, and anti-WFS1 antibody and reprobed with anti-Calnexin antibody (**D**) The densities of proteins bands are measured, and the ratio is calculated (**E**–**G**). Each column represents mean ± SEM. Data was analyzed using one-way ANOVA followed by Tukey’s post hoc test; ^*^P < 0.05, ^**^P < 0.01, ^***^P < 0.001 versus CTRL group; ^+^P < 0.05, ^++^P < 0.01, ^+++^P < 0.001 versus PPPLS group; ^ɛ^P < 0.05, ^ɛɛ^P < 0.01 versus PLS group; ^δ^P < 0.05, ^δδ^P < 0.01 versus PPLS group. *CTRL* non stress, *PPPLS* pre-pregnancy, pregnancy, lactation stress, *PPS* pre-pregnancy stress, *PS* pregnancy stress, *LS* lactation stress, *PPPS* pre-pregnancy, pregnancy stress, *PLS* pregnancy, lactation stress, *PPLS* pre-pregnancy, lactation stress.

Statistical analysis revealed that Bip mRNA expression increased in the pancreatic tissue of PPPLS, LS and PPLS, PPS, PLS, PS, and PPPS groups compared to CTRL group (P < 0.001 to P < 0.05). Bip mRNA expression levels in PPPS groups were significantly higher than that of PPPLS group (P < 0.01); however, the expression of Bip mRNA in PLS group reduced compared to PS group (P < 0.05, Fig. 5A).

In the extracted ER of pancreas, Bip protein level in all stress groups except PPPLS were significantly higher than that of CTRL group (P < 0.001 to P < 0.05). Moreover, this parameter in PPPS groups was significantly higher than that of PPPLS group (P < 0.001). There were significant differences between Bip protein levels in pancreatic ER of PPS and PS groups compared to those of PPLS and PLS groups respectively (P < 0.01 to P < 0.05, Fig. 5D,E).

### Chop mRNA and protein expression levels

According to ANOVA analysis the pancreatic Chop mRNA expression levels in all stress groups except PPPLS and PPLS groups were significantly elevated compared to CTRL group (P < 0.001 to P < 0.05). Moreover, Chop mRNA expression levels in PPPS groups were significantly higher than that of PPPLS group (P < 0.001). This parameter in PS group increased compared to PLS group (P < 0.05, Fig. 5B).

Chop protein levels in the extracted ER of pancreas of PPS, LS, PLS, PS, and PPPS groups were significantly higher than that of CTRL (P < 0.001 to P < 0.05); however, there was not any significant differences between the pancreatic Chop protein levels of PPPLS, PPLS and CTRL groups. Chop protein level in pancreas of PS group significantly increased compared to those of PLS groups (P < 0.05). This parameter in PPPS group elevated compared to those of PPPLS groups (P < 0.001, Fig. 5D,F).

### WFS1 mRNA and protein expression levels

Statistical analysis revealed that the pancreatic WFS1 mRNA level in all stress groups significantly increased compared to CTRL group (P < 0.001 to P < 0.05). WFS1 mRNA expression in pancreas of PPPS groups was significantly higher than that of PPPLS group (P < 0.05). This parameter in PS group also elevated compared to PLS group (P < 0.01, Fig. 5C).

In the extracted ER of pancreas, WFS1 protein level in all stress groups except PPPLS and PPLS groups was significantly higher than that of CTRL group (P < 0.001 to P < 0.05). This parameter in PPPS groups elevated significantly compared to PPPLS group (P < 0.001). Moreover, WFS1 protein level in the pancreatic extracted ER of PPS and PS groups significantly increased compared to those of PPLS and PLS groups respectively (P < 0.01, Fig. 5D,G).

### GR mRNA and protein expression levels

**Figure 6:**
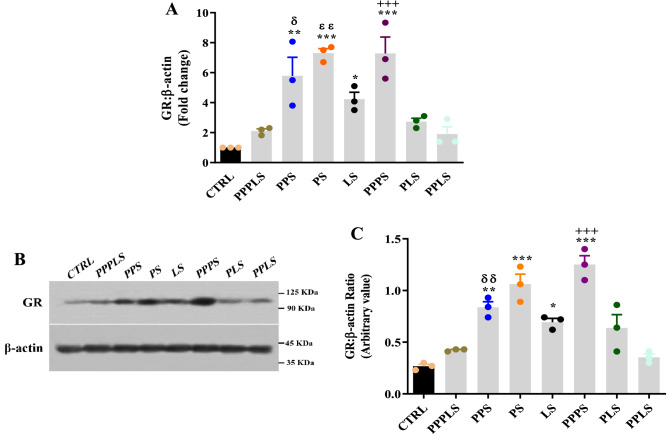
Effect of perinatal stress on pancreatic GR (**A**) protein (**B**, **C**) levels in the young adult male offspring. Each column represents mean ± SEM (3 rats/group, 3 litters/group). Data was analyzed using one-way ANOVA followed by Tukey’s post hoc test; ^*^P < 0.05, ^**^P < 0.01, ^***^P < 0.001 versus CTRL group; ^+++^P < 0.001 versus PPPLS group; ^ɛɛ^P < 0.01 versus PLS group; ^δ^P < 0.05, ^δδ^P < 0.01 versus PPLS group. *CTRL* non stress, *PPPLS* pre-pregnancy, pregnancy, lactation stress, *PPS* pre-pregnancy stress, *PS* pregnancy stress, *LS* lactation stress, *PPPS* pre-pregnancy, pregnancy stress, *PLS* pregnancy, lactation stress, *PPLS* pre-pregnancy, lactation stress.

The expression of GR mRNA in the pancreatic tissue of PPS, PS, PPPS, and LS groups elevated significantly compared to that of CTRL group (P < 0.001 to P < 0.05). This parameter in PPPS groups was significantly higher than that of PPPLS group (P < 0.001). Pancreatic GR mRNA expression in PPS and PS groups increased significantly compared to PPLS and PLS groups respectively (P < 0.01 to P < 0.05, Fig. 6A).

Statistical analysis revealed that GR protein of PPPS, PS, LS and PPS groups significantly elevated compared to CTRL group (P < 0.001 to P < 0.05). This value in PPPS groups was significantly higher than that of PPPLS group (P < 0.001). Moreover, this parameter in PPS groups increased compared to that of PPLS group (P < 0.01, Fig. 6B,C).

### WFS1 DNA methylation

**Figure 7:**
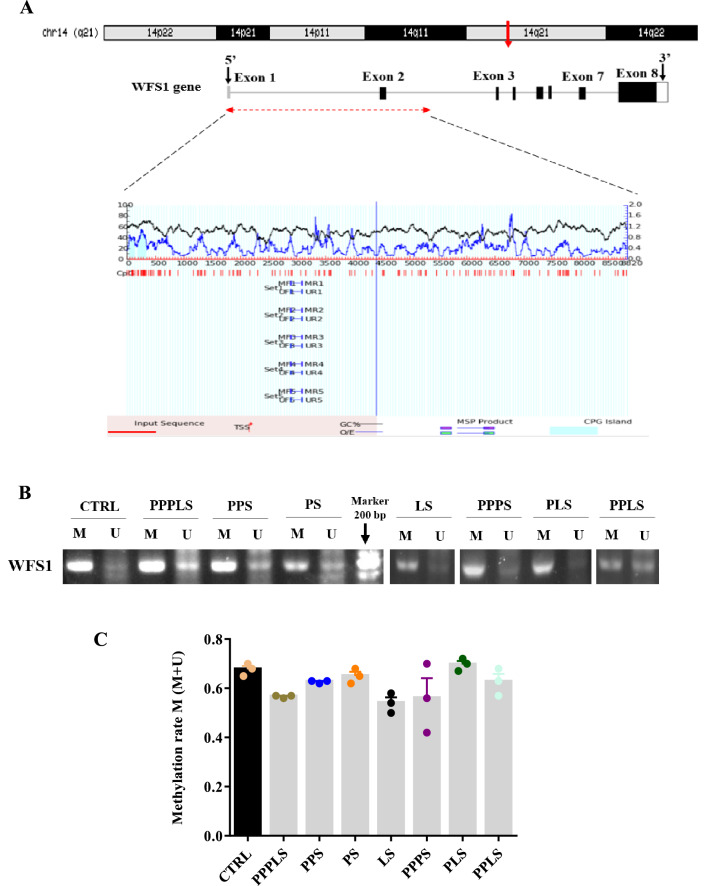
Effect of perinatal stress on DNA methylation of WFS1 gene in pancreatic tissue. Schematic presentation of promoter area of the WFS1 gene encompassing investigated CpG Island (**A**). Agarose gel electrophoresis demonstrating the methylation status of WFS1 promoter in pancreatic tissue by methylation-specific PCR (MSP). Primer sets used for amplification are designated as un-methylated (U), and methylated (M) (**B**). Each column represents mean ± SEM (3 rats/group, 3 litters/group). *CTRL* non stress, *PPPLS* pre-pregnancy, pregnancy, lactation stress, *PPS* pre-pregnancy stress, *PS* pregnancy stress, *LS* lactation stress, *PPPS* pre-pregnancy, pregnancy stress, *PLS* pregnancy, lactation stress, *PPLS* pre-pregnancy, lactation stress.

As shown in Fig. 7A–C, there was not any significant changes in the methylation levels of WFS1 gene in the pancreas of different groups.

## Discussion

**Figure 8:**
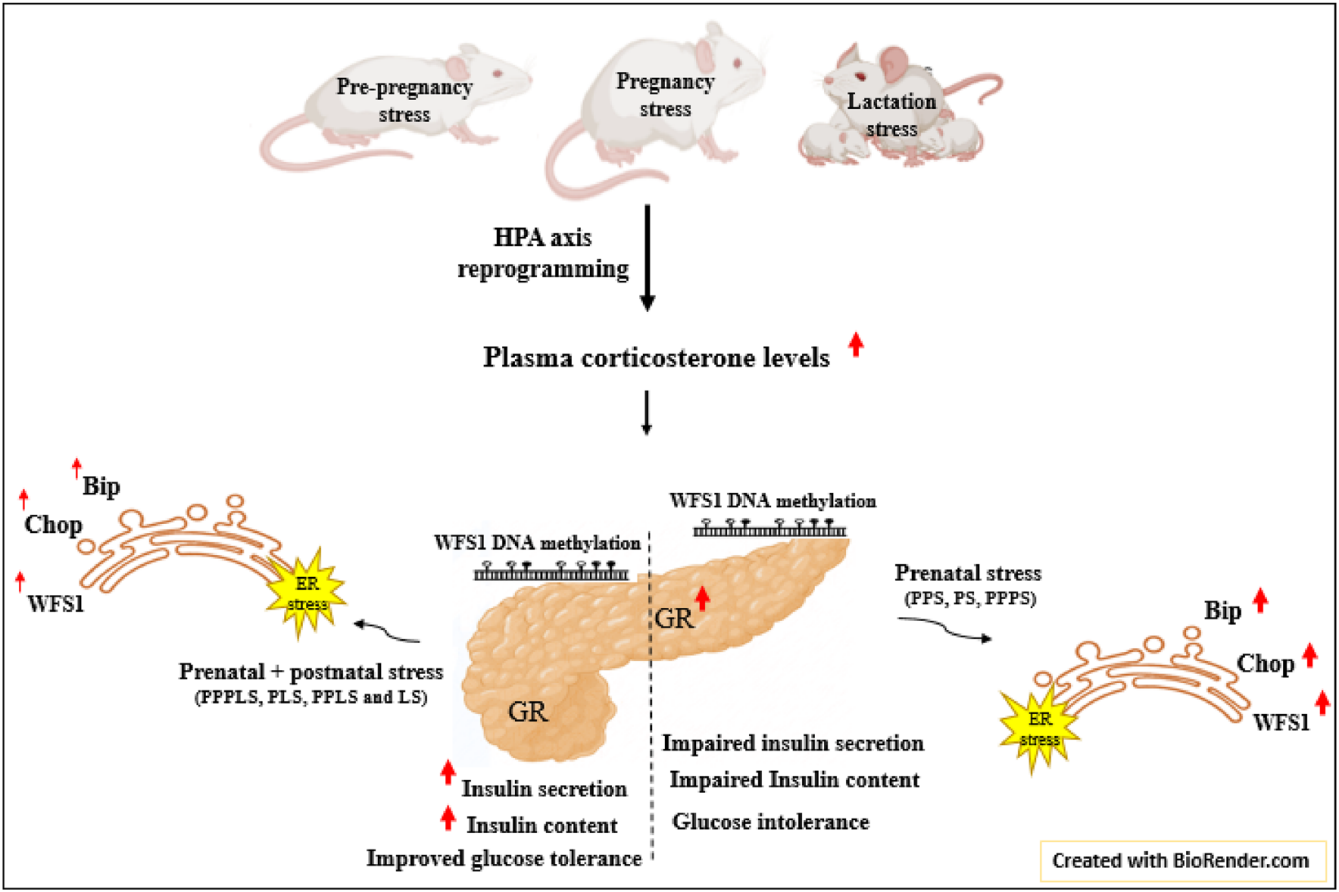
Summary of conclusion.

The current study evaluated the hypothesis that exposure to high corticosterone level induced by perinatal stress could affect the programming of metabolic systems in offspring. The findings of this investigation demonstrated that the offspring who exposed to prenatal along with postnatal stress (in PPPLS, PLS, PPLS and LS groups) showed marginal increase of the pancreatic tissue mRNA and pancreatic extracted ER protein levels of Bip, Chop and WFS1. While they showed a marked increase of isolated islets insulin secretion and content. On the other hand, despite the sharp increment of pancreatic tissue mRNA and pancreatic extracted ER protein levels of Bip, Chop and WFS1, there were no significant changes in insulin secretion and content of isolated islets in the offspring who only exposed to prenatal stress (in PPS, PS, and PPPS groups). Moreover, exposure to prenatal or postnatal stress (i.e. in PPS, PS, LS and PPPS groups) increased mRNA expression and protein levels of GR in pancreatic tissue. Interestingly, we demonstrated for the first time that DNA methylation of WFS1 gene did not change in adult rat offspring of the stressed groups. Furthermore, maternal stress impaired glucose tolerance, and significantly increased basal plasma corticosterone and insulin levels as well as HOMA-IR index, however, decreased ISI _Matsuda_ (Fig. 8).

In the current study, baseline plasma corticosterone levels at the first and last days of exposure to variable stress throughout perinatal periods significantly enhanced in the dams of the stressed groups; therefore, we were able to examine the effect of each period of stress alone or in combination on offspring glucose metabolism. Similar to our results, the multiplicity of stressors as used in this study, did not lead to corticosterone response adaptation in the dams who repeatedly exposed to stress^24^; however, some studies have reported reduced plasma corticosterone level following maternal stress in dams^25^. It is considered that the different plasma corticosterone levels could be related to the alternation of HPA axis sensitivity induced by exposure to repeated stress. In the offspring of the stressed groups except LS and PPLS groups, plasma corticosterone level on PND1 did not change; however, it increased on PND21 compared to the control offspring. In contrast, studies demonstrated that exposure for 24 h to a sound stress on 10th-18th days of gestation resulted in a non-significant reduction of basal corticosterone level in C57Bl/6 mice on PND1^26^. Brummelte et al. reported that injection of exogenous corticosterone during pregnancy (10th–20th gestational days) and postpartum (2nd–21st days) increased serum corticosterone levels on PND1, but did not affect its levels on PND21 in rat offspring^27^. These differences between studies may be due to several possible mechanisms. In this regard, researchers showed that fetus access to maternal corticosterone is low because of the action of the placental enzyme11β-HSD2, which transforms maternal corticosterone to inactive cortisone but this barrier can be further weakened by maternal stress^27^. Maternal plasma corticosterone-binding globulin (CBG) levels may also have a role in the regulation of plasma corticosterone levels available to the fetus, and prenatal stress can decrease maternal CBG levels^28^. Previous study indicated that maternal CRH released by the hypothalamus during stress could transfer across the placenta and activate the HPA axis in the rat offspring^29^. On the other hand, increased maternal catecholamine levels could lead to hypoxia-induced vasoconstriction of the placental vessels^30^, which in turn activated the fetal HPA axis. According to the above-mentioned studies, exposure to perinatal stress could increase the transfer of maternal corticosteroids directly or indirectly across the placenta to the fetus. Therefore, it is possible that the offspring has been adapted to stress which could explain the unchanged plasma levels of corticosterone at PND1. The increase in serum corticosterone level on PND21 might, at least partially, be due to a direct transfer of maternal corticosterone to offspring through the milk. The baseline corticosterone levels in the adult offspring of stress groups except PPS group, were higher than those of controls. In this respect, animal studies indicated that exposure to prenatal noise stress associated with physical stressors on days 12th–16th of gestation in the C57BL/6 mice caused the elevation of HPA-axis function in offspring^31^. Several animal studies have shown that inducing prenatal stress in offspring by maternal exposure to chronic unpredictable stress (CUMS) during pregnancy^24^, and injection of corticosterone to adrenalectomized dams during prenatal period^32^ could increase the activity of the HPA axis and the plasma corticosterone levels in rat offspring. Moreover, a more recent study indicated that exposing dams to chronic social defeat stress (CSDS) before pregnancy (preconception stressors) for 3 weeks activated HPA axis and increased the basal corticosterone levels in their adult offspring^33^. Together these results suggest that exposure to high maternal corticosterone during perinatal period probably affect the programming of the HPA axis in the offspring which is dependent on the stressor type, intensity of stress, time and/or duration of stress exposure as well as the age of offspring at study.

Our results demonstrated that maternal stress-exposed offspring had increased plasma insulin levels and insulin resistance (HOMA-IR index), and decreased plasma glucose levels and insulin sensitivity (Matsuda index) in adulthood. Studies reported that exposure to psychological stress during pregnancy in humans^34^ and chronic unpredictable mild stress during last week of pregnancy elevated plasma corticosterone levels and HOMA-IR index in rat offspring^35^. Previous animal research showed that maternal exposure to cortisol (0.48 mg/h) during early pregnancy did not alter insulin sensitivity in adult male sheep offspring^36^. Moreover, karbaschi et al. demonstrated that maternal high fat diet as a psychophysical stressor during pregnancy and lactation periods increased plasma corticosterone levels and insulin sensitivity in adult rat offspring^37^. In general, maternal stress and exposure to elevated plasma corticosterone levels in different models may program insulin resistance and insulin sensitivity in the offspring which may be attributed to alterations in insulin receptor signaling pathway in peripheral tissues. For instance, offspring exposed to maternal stress showed reduced expression of insulin receptor (INSR) and insulin receptor substrate 1 (IRS1)^38^, AKT protein levels (total and activated/phosphorylated forms)^39^, expression and/or translocation of Glut4 in the muscle, liver and adipose tissues^40^, which were the mechanisms of insulin-dependent glucose uptake and utilization. Therefore, it was possible that perinatal stress could alter HOMA-IR and ISI indices by interfering with these mentioned pathways.

Our findings indicated that although maternal stress is able to impair glucose-insulin homeostasis in adulthood, glucose loading, could improve glycaemia (except in the PS group) during perinatal period. In line with our results, Blasio et al. reported that exposure to excess maternal glucocorticoids in early pregnancy period led to hyperinsulinemia, and improved glucose tolerance in adult male sheep offspring^36^. In contrast, the adult male rat offspring whose dams exposed to social stress during late pregnancy showed an unchanged plasma glucose or insulin concentrations following glucose tolerance test^41^. On the other hand, studies have revealed that exposure to dexamethasone^10^, as well as administration of carbenoxolone as inhibitor of placental 11 beta-hydroxysteroid dehydrogenase type 2 (11β-HSD2) during pregnancy resulted in hyperglycemia, hyperinsulinemia, and impaired glucose tolerance in 6-months old rat offspring^42^. Therefore, it was possible that impaired glucose-insulin homeostasis in the adult offspring of the present study resulted partly from programming of insulin signaling pathways by an adverse maternal environment and excess corticosterone levels. Increased insulin secretion reflects a compensatory mechanism for observed insulin resistance which may explain improved glucose tolerance in the offspring^43^**.** Moreover, in PS group glucose tolerance was impaired, possibly due to impaired glucose-stimulated insulin secretion.

In the current study, there were significant elevation in the pancreatic GR mRNA and protein levels of adult offspring who exposed to prenatal or postnatal stress (i.e. in PPS, PS, LS and PPPS groups), whereas no changes were seen in offspring of mothers exposed to prenatal plus postnatal stress (i.e. PPPLS, PLS and PPLS groups). To our knowledge, there are not any studies in subject of the effects of perinatal stress on GR expression in pancreatic tissue. However, animal studies have revealed that prenatal dexamethasone administration in the last week of pregnancy increased^44^ or decreased^45^ GR mRNA expression and protein levels in liver, muscle and adipose tissues in the adult offspring. In another study in mice offspring, maternal exposure to restraint stress during pregnancy elevated GR gene expression and protein levels in liver tissue^46^. In contrast, Brunton et al. exposed the rat dams to aggressive rat as a psychosocial stress during late gestation period and reported a reduction of GR mRNA expression in muscle tissue in their adult offspring^41^. The above-mentioned studies showed that the levels of GR expression in different peripheral tissues were altered by an adverse maternal environment. It seems that exposure to excessive postnatal care (LS period) could result in the increased GR mRNA and protein expression in the PPS, PS, LS and PPPS groups. In this regard, studies indicate that the GR expression level is affected by the level of maternal care^47^. On the other hand, recent findings have suggested the involvement of the epigenetic modifications (methylation/demethylation), which were induced by early life adverse environment in the specific promoters of the GR gene. These epigenetic modifications could permanently change the programming of GR gene expression^48^. Howbeit, we did not assess epigenetic programming of GR in the pancreas of offspring in this investigation.

Our study also indicated that offspring who exposed to prenatal stress (i.e. in PPS, PS, and PPPS groups) had markedly increment in Bip, Chop and WFS1 protein and mRNA levels in both pancreatic tissue and its extracted ER, but no change was seen in insulin secretion and insulin content of the isolated pancreatic islets in response to basal (5.6 mM) and high (16.7 mM) glucose concentrations in these groups. On the other hand, offspring who exposed to prenatal plus postnatal stress (i.e. in PPPLS, PLS, PPLS, and LS groups) had marginal increase of Bip, Chop and WFS1 protein levels in the extracted ER of pancreas and these changes were accompanied with the increased insulin secretion and insulin content of the isolated pancreatic islets in response to basal and high glucose concentrations. The recent study has determined that short exposure to low-dose endogenous corticosterone in 12-weeks old C57BL6 mice caused ER stress via the glucocorticoid receptor and directly increased the levels of Bip, XBP1 and ATF6 at both mRNA and protein levels in macrophage cells, whereas a longer exposure to a higher dose did not affect macrophage function^15^. Other study showed that exposure to prednisolone induced a GR-mediated impairment in ER homeostasis which led to increased activation of UPR signaling pathways, inhibition of insulin biosynthesis and glucose-stimulated insulin secretion in the INS-1E cells^14^. The previous study reported that WFS1 is an ER membrane-embedded protein, which can be up-regulated under ER stress and also plays an essential role in insulin expression and secretion. For instance, a study reported that exposure to ER stress-inducers (thapsigargin and dithiothreitol), caused the elevation of WFS1 mRNA and protein levels in mouse β-cell line, MIN6 cells^49^. A recent study by Damien Abreu showed that defects of WFS1 in vivo and in vitro led to ER stress and reduced insulin secretion and content in response to glucose, and triggered Chop-Trib3 axis in WFS1-knockout mice^50^ According to the above-mentioned findings, most of the studies on ER stress markers have been done in human and animal cell lines and also WFS1 gene studies have been performed mainly in knocked out animal models. In our study, for the first time, the function of WFS1 and UPR in offspring were examined specifically in the pancreatic extracted ER. Our results showed that exposure to maternal stress can lead to impaired WFS1 expression level and its function as a regulator of ER calcium channels activity in offspring, which it may be the cause of insulin secretion and insulin content impairment in later life**.** In accordance with these findings, Kakiuch reported that WFS1 forms a complex with ER chaperone glucose-regulated protein 94 (GRP94) in neuronal cells. Therefore, down-regulation of GRP94 may increase the amount of GRP94-free WFS1, leading to the enhancement of WFS1 function^51^. The studies in humans and animal models have shown that maternal exposure to synthetic glucocorticoids during pregnancy could lead to epigenetic alterations in the offspring DNA which resulted in programming changes and long-lasting effects on the expression of some genes^52^. Thus, in the present study, it is possible that perinatal stress caused epigenetic changes in the UPR signaling pathway in offspring pancreatic islets, which needs further investigation. However, in current study, for the first time, the effect of perinatal exposure to stress evaluated on the methylation of CpG islands at promoter regions for WFS1 gene. The results showed that perinatal stress did not affect DNA methylation level of WFS1 gene in different study groups. There is now extensive evidence that part of the effects of adverse experiences in the critical period of life alteration corticosterone gene expression^47^. Thus it seems that in the PPS, PS, and PPPS groups, the elevated levels of the plasma corticosterone and pancreatic expression of mRNA and protein of GR could contribute in GR-mediated impairment of ER homeostasis, so the complex between WFS1 and ER chaperone probably became stronger and WFS1 remained in the ER to maintain or restore ER homeostasis which lead to disturbances in glucose metabolism in these stressed groups. Histological analysis at the tissue level revealed that WFS1 was expressed not only in ER but also in secretory granules to regulate acidification and maturation of insulin protein in mouse pancreatic β-cells^53^.

In this study, the dams were inevitably deprived of water and food during exposure to stress (for 60 min). It should be noted that deprivation of water and food is also considered as a stressor and in many studies that use the variable stress paradigm, it is considered as one of the stressors^54,55^. As mentioned above, water and food deprivation is an inevitable topic in our study. However, considering that the animals were exposed to stress during light phase, in which the animals are not active in terms of feeding^56^, and also regarding that this type of stressor has a psychological aspect, like other types of stressors used in this study, water and food deprivation may not have a profound negative effect on the results. Moreover, According to previous studies, from weaning, the male offspring were housed in same-sex groups of three animals per cage to the end of the experiment^57^, thus a state of adaptation was established between rat animals and with monitoring the animals’ behavior, there was no state of dominancy in rats to affect the study results also, food intake was measured by calculating the difference between fixed amount of food and the amount remaining after 24 h. this amount was then divided by 3 and was considered as food intake for each animal because there were no significant difference between the animals’ body weight therefore the effect of food competition between animals on the metabolic changes may be weak. Based on the results of the present study, it seems that the probable increment of maternal care during lactation could reduce the effects of maternal stress on the offspring^58,59^ therefore, stress-related changes in the offspring metabolic systems were mainly observed during pre-pregnancy and pregnancy periods. These results showed the effect of maternal care on the level of gene expression in metabolic and neuroendocrine systems of offspring, which needs further investigation^9,60,61^. The data of the present study provide new insight about effects of WFS1 on glucose-insulin homeostasis, particularly in the context of stress during critical periods of development, therefore these findings provide motivation for further research studies and also therapeutic approaches targeting WFS1 as a treatment for stress-related metabolic disorders in later life.

## Conclusions

Findings of the current investigation showed that prenatal maternal stress affected glucose metabolism in adult male rat offspring through the induction of ER stress and the increased pancreatic GR expression, as well as increased pancreatic extracted ER WFS1 protein expression. Which, in turn led to the impairment of glucose-stimulated insulin secretion and insulin content. These alterations seems to be compensatory responses to preserve ER homeostasis.

## Limitations of study

Based on the results of this study, the following suggestions are made: investigating the epigenetic changes of glucocorticoid receptor in the pancreatic tissue, assessment of intracellular Ca^2+^ level in pancreas tissue, determining the levels of apoptotic factors regulated by the UPR signaling pathways in pancreas tissue relative to pancreatic beta-cells death.

## Materials and methods

### Animals

Male and female Wistar rats (10–12 weeks old, 200–250 g) were kept in a temperature-controlled room (22 ± 2 °C) with a 12:12-h light: dark cycle and their food (standard pellets, Pars Company, Tehran, Iran) contained 2 g% soybean oil, which provided 4.75% Kcal as fat; 17.5 g% protein, which provided 18.48% Kcal; 72.72 g% carbohydrates, which provided 76.77% Kcal and water were provided ad libitum (it is noteworthy that experimental groups were deprived of food and water in the testing days). Mating occurred overnight with two females and one male in a cage, pregnancy was confirmed based on the presence of sperm in the vaginal smear on the following morning and considered as gestational day 0 (GD0). The methods of the present study has been reported in accordance with ARRIVE guidelines^62^. All experimental procedures were performed according to the previously published guideline for the Care and Use of Laboratory Animals (NIH Publication No. 85-23, revised 1996) and approved by the Ethics Committee of the physiology Research Center, Tabriz University of Medical Sciences, Tabriz, Iran (IR.TBZMED.REC.1397.336) and Ethics Committee of Shahid Beheshti University of Medical Sciences, Tehran, Iran (IR.SBMU.MSP.REC.1400.300).

### Experimental design

**Figure 9:**
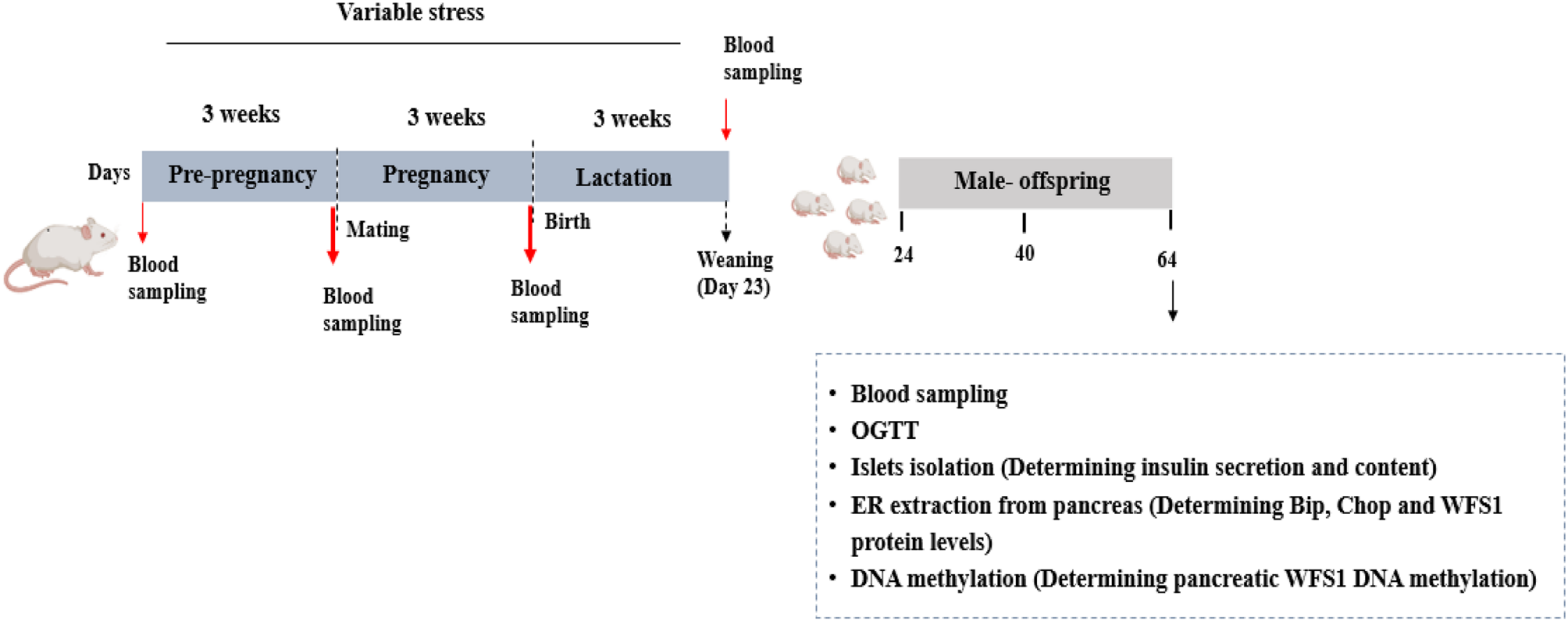
Experimental procedures over the course of the experiment.

Dam rats were assigned to the experimental groups consisting of seven stress groups and control group. The dams were exposed to variable stress for 21 consecutive days before mating (during pre-pregnancy period), or/and during pregnancy and lactation periods. After delivery, the pups of each group were kept with their mothers. Table 1 showed the litter size, the percentages of male puppies per litter and death in each group. All experiments were done at postnatal day (PND) 64 (28 rats/group). Figure 9 depicted the experimental design of this study.

**Table 1:** Effects of variable stress on litter size, percent males, and death rate.

Groups	Litter size (pups)	% Male (pups/litter)	% Death rate
CTRL	11.50 ± 0.65	57.80 ± 6.34	9.20 ± 4.12
PPPLS	9.75 ± 0.86	46.18 ± 8.27	15.67 ± 6.96
PPS	11.00 ± 0.55	49.23 ± 4.38	3.64 ± 2.23
PS	11.40 ± 0.75	49.98 ± 4.83	8.89 ± 7.26
LS	9.80 ± 0.63	54.90 ± 11.86	11.67 ± 7.26
PPPS	11.24 ± 0.58	39.29 ± 3.65	2.00 ± 2.15
PLS	10.00 ± 0.37	46.67 ± 6.94	8.04 ± 3.76
PPLS	8.83 ± 0.31	43.06 ± 7.45	2.22 ± 2.50

Following the first and last exposure to stress, blood samples of dams and offspring (PND-1 and PND-21) were taken using retro-orbital-puncture method to measure plasma corticosterone levels. They were weaned on PND-23, then male pups of each litter (There are sex differences in the occurrence of metabolic disorders compared to females, and males are more susceptible to the metabolic disturbances induced by environmental challenges^63^, therefore this study examined male rat offspring to indicate the metabolic disturbances due to maternal stress) were randomly assigned into eight groups according to the maternal stress as follows: CTRL group; the offspring of control mothers who were not exposed to any stresses, PPPLS group; the offspring of mothers who were exposed to stress during pre-pregnancy, pregnancy and lactation periods, PPS group; the offspring of mothers who were exposed to stress during pre-pregnancy period, PS group; the offspring of mothers who were exposed to stress during pregnancy period, LS group; the offspring of mothers who were exposed to stress during lactation period, PPPS group; the offspring of mothers who were exposed to stress during pre-pregnancy and pregnancy periods, PLS group; the offspring of mothers who were exposed to stress during pregnancy and lactation periods, PPLS group; the offspring of mothers who were exposed to stress during pre-pregnancy and lactation periods. From weaning, the male offspring were housed separately (three per cage) and had free access to a normal diet until experimental day. At young adulthood (PND-64), in the offspring of all study groups, following an overnight fasting blood samples were taken to measure plasma glucose and insulin levels as well as HOMA-IR and Matsuda indices, then oral glucose tolerance test (OGTT) was performed. Finally, the animals were decapitated to remove their pancreas in order to islet isolation and assessment of glucose-stimulated insulin secretion along with insulin content, WFS1, Bip, Chop, and GR mRNA and protein levels and DNA methylation of WFS1 gene as well as WFS1, Bip and Chop protein levels of rough endoplasmic reticulum.

### Stress procedure

The dams of different stress groups were subjected to variable stress during specific periods according to their grouping. The variable stress paradigm was used to prevent the possible habituation to a repeated homotypic (same) stressor. The stressors were applied during the light cycle at different times of the day (at 8–10 AM or 14–16 PM). All stressors used are known to induce endocrine responses in male rats. Moreover, because the duration of exposure to stress in this study were long (3 weeks pre-pregnancy, 3 weeks pregnancy, and 3 weeks lactation periods), so we have applied psychological stress with different intensities that dam can tolerate and also do not adapted to it. The stressors were as follows: (1) transparent plexiglass cylinder (9.5 × 20 cm, 60-min); (2) wire mesh restrainer (4 × 8 cm, 60-min); (3) decapiCone Restrainer (small plastic bag with a hole in the end that only the rat’s head can fit through, 60-min); (4) social stress (move the animals to a new cage with unfamiliar animals, 60-min); (5) swimming stress (transparent tank, 18 × 40 cm, containing 12 cm of water 23 ± 2 °C, 15-min); (6) shaking (high speed, 20-min); (7) nip tail (1/3 tail terminal, 10-min); (8) bright light (three times, 100 W lamp, 10-min)^54,64,65^.

### Blood sampling

Blood samples of dams were collected after the first and the last exposures to stress and also adult offspring of each group on PND64 using retro-orbital puncture method. The offspring were decapitated on PND1 and their trunk blood were collected. In all blood sampling, blood was collected following isoflurane (6.5 ml/l/kg of isoflurane/desiccator volume/rat body weight) (Baxter, USA)^66^ anesthesia after overnight fasting. The blood samples were collected in heparinized (5000 IU/mL, Caspian Tamin, Rasht, Iran, 10 µL/mL) micro tubes and centrifuged at 664 × g at 4 °C for 10 min. Then, the plasma was stored at –80 °C until corticosterone measurement. Plasma levels of corticosterone were measured using the rat corticosterone ELISA kit (ZellBio GmbH, Ulm, Germany). (Minimum detection: 0.9 nmol/l). Intra- assay coefficients of variation (CVs) were 6.4%.

### Oral glucose tolerance test (OGTT)

After overnight fasting on PND64, a bolus of glucose solution (%20 in distilled water, 2 g/kg body weight) (Merck, Germany) was given to adult rats by oral gavage and blood samples were collected at 30, 60, and 120 min after glucose load in order to evaluate plasma glucose and insulin concentrations^67^. Plasma glucose and insulin levels were determined using glucose oxidase method (Pars Azmoon Co., Tehran, Iran) and enzyme-linked immunosorbent assay method (Rat insulin ELISA kit; ZellBio GmbH, Ulm, Germany) respectively. (Minimum detection: 1 mg/dl and 0.1 mIU/L respectively). Intra- assay coefficients of variation (CVs) were 2.1%, and 5.1% respectively.

### Calculation of HOMA-IR and matsuda indices

Insulin resistance was assessed in the fasting state by calculating homeostasis model assessment according to the following formula:

HOMA-IR = Fasting glucose (mmol/l) * Fasting insulin (µU/ml)/22.5

Insulin sensitivity was calculated using all GTT time points in the following formula:

ISI (Matsuda) = 10,000 /√ [(G_fasting_ × I_fasting_) × (G_OGTT mean_ × I_OGTT mean_)]

Where G_fasting_ is fasting plasma glucose expressed as mg/dl, I_fasting_ is fasting plasma insulin expressed as μU/ml, G_OGTT mean_ is mean plasma glucose concentration after glucose loading and I_OGTT mean_ is the mean insulin concentration after glucose loading^68^.

### Islet isolation procedure

The islet isolation was performed by the collagenase technique of Lacy and Kostianovsky (1967)^69^ with slight modification^70^ (Please see the details in Supplementary Method).

### Isolated islets’ Glucose-stimulated insulin secretion and content

Glucose-stimulated insulin secretion and insulin content was assessed at 5.6 and 16.7 mM glucose concentrations^70^ (Please see the details in Supplementary Method).

### Isolation of rough endoplasmic reticulum (RER)

Rough vesicles derived from RER of rat pancreatic cells were extracted by the slightly modified method^71^ described by Kan et al. (1992). Briefly, the rats of each group (8 rats/group) were anesthetized by isoflurane and decapitated. Then, their pancreases were removed and homogenized in 30 ml of an ice-cold sucrose (0.25 M) solution at 630.8 × g using electric potter homogenizer (Potter–Elvehjem Homogenizer, Iran). The homogenate was then filtered through surgical filter made of fabric cotton. By adding ice-cold sucrose (0.25 M) solution, the homogenate reached a volume of 60 ml, and it was centrifuged at 1881.33 × g for 13 min. Subsequently, the supernatant was centrifuged at 4957.87×*g* for 14 min, and at 7923.73 × g for 67 min, at 4 °C (Beckman model J-21B, USA). The pellet was dissolved in 9 ml of ice-cold sucrose (2 M), and transferred to a glass homogenizer to be manually homogenized for 20–25 times. Subsequently, the obtained suspension was centrifuged at 12,129.07×*g* for 67 min in a sucrose gradient condition (including 1 M and 2 M sucrose solution) and the resulting pellet was dissolved in 20 ml sucrose imidazole pyrophosphate (0.25 mM sucrose, 3 mM imidazole, 0.5 mM Na pyrophosphate). Then it was centrifuged at 8941.87×*g* for 47 min and repeated for three times. Finally, the resulting pellet (RER vesicles) was dissolved in 1 ml sucrose imidazole (0.25 mM sucrose, 3 mM imidazole) at a final concentration of 7 mg/ml and stored in 10 µl aliquots, pH 7.4, at − 80 °C until being used.

### Measurement of protein levels in pancreatic rough endoplasmic reticulum (RER) and pancreatic tissue

Protein levels were analyzed by Western blotting (Please see the details in Supplementary Method). In this study the following primary antibodies were used; WFS1#26110; St John’s Laboratory, Bip #21685; Abcam, Chop #11419; Abcam, GR #223138; Abcam, β-actin #8226; Abcam, Calnexin #22595; Abcam.

### Measurement of Bip, Chop, WFS1, GR mRNA levels in pancreatic tissue

In order to determine mRNA levels of Bip, Chop, WFS1, and GR in pancreatic tissue, quantitative real-time PCR (QRT-PCR) method was used. The primer sequences are shown in Table 2 (Please see the details in Supplementary Method).

**Table 2:** Primers used for real-time PCR analysis.

Primer name	Gene bank accession no	Primer sequence (5′→3ʹ)
WFS1	NM_ 031,823.1	Forward: GACAAGATTGAACCGCCTCGT
		Reverse: CCACATCTGCCTTCATGGGAC
Bip	NM _ 013,083.2	Forward: CAATGACCAAAACCGCCTGA
		Reverse: GCTTTCCAATTCATTCCTCGT
Chop	NM_001109986.1	Forward: ATGAACTGTTGGCATCACCT
		Reverse: TGCACTGGAGATTACATGCTT
GR	NM_012576.2	Forward: AGGCTTCAGAAACTTACACC
		Reverse: CAAATGCCATGAGAAACATCC
ß-Actin	NM_31144.3	Forward: CTCATGAAGATCCTGACCGAG
		Reverse: TCTCTTTAATGTCACGCACGA

*WFS1* wolframin ER transmembrane Glycoprotein, *Bip (Hsp70)* heat shock protein family A member 4, *Chop (Ddit3)* DNA-damage inducible transcript3, *GR (Nr3c1)* nuclear receptor subfamily 3, group C, member 1.

### DNA methylation of the WFS1 gene promoter in pancreatic tissue

Methylation-specific PCR (MSP) method was performed for assessing methylation differences at specific CpG sites WFS1 gene based on bisulfite treatment of DNA^72^.

### Extraction and bisulfite modification of DNA

Genomic DNA from pancreatic tissue was extracted using the phenol–chloroform method. Briefly, 300 μl of lysis buffer (containing: Sucrose, 0.32 M; Tris, 10 mM; MgCl_2_, 5 mM; and SDS, 1%) was added to the tissue and incubated at 40 °C for 20 min, then 300 μl of phenol solution and chloroform were added. After centrifugation at 1770.67 × g for 5 min, the supernatant was transferred to a clean tube containing acetate sodium 0.3 M and absolute alcohol (Merck, Germany). Then, the DNA solution was incubated at − 20 °C for 2 h. After a further centrifugation step (30 min at 2877.3 × g) and adding 100 μL 70% EtOH, the DNA tube was air-dried and resuspended in 30 μL ddH_2_O. The concentration and purity of DNA were determined using Nano drop spectrophotometer (Thermo Fisher Scientific, USA). The DNA was modified by bisulfite using the EpiTect Bisulfite Conversion kit (QIAGEN), according to the manufacturer’s protocol. Briefly, 500 ng DNA (500 ng/ml) was mixed with 85 µL bisulfite mix, 35 µL DNA protect buffer and adjusted to a volume of 140 μL with nuclease-free water. The following cycling conditions were used: 95 °C for 5 min, 60 °C for 25 s, 95 °C for 5 min, 60 °C for 85 min, 95 °C for 5 min, and 60 °C for 175 min. Then, the modified DNA was washed repeatedly with buffers (BW, BD, BL, and EB) and subsequently centrifuged at 2656×*g* for 1 min, finally it stored at − 20 °C.

### Methylation-specific PCR (MSP)

Methylated (M) and unmethylated (U) sets of primer pairs for MSP were designed using the Meth Primer software, the primer sequences are listed in Table 3. The amplification conditions were 95 °C for 5 min (initial denaturation) followed by 40 cycles of 95 °C for 30 s (denaturation), 58 °C for 30 s (annealing), 72 °C for 30 s (extension); and 7 min as final extension at 72 °C. All PCR reactions were carried out using a BIOER Thermal Cycler 9500 (Hangzhou Bioer Technology Co., Ltd., Binjiang, China). PCR products were resolved by electrophoresis on 2% agarose gels. In order to determine the degree of methylation of WFS1 gene, the Image J software was used and the intensities of bands were assessed.

**Table 3:** Primers used for MSP methylation.

Primer name	Product size (bp)	Primer sequence (5′→3ʹ)
WFS1 methylation (M)	199	Forward: GCGTTTATAGGGAAAGAATGTC
		Reverse: ACACAAAACACTAAAAACTTCACGAC
WFS1 unmethylation (U)	200	Forward: GGTGTTTATAGGGAAAGAATGTT
		Reverse: ACACAAAACACTAAAAACTTCACAACT

### Data analysis and statistics

The data were shown as mean ± standard error of the mean (SEM). One-way analysis of variance (ANOVA) (by considering stress as an independent factor) and two-way repeated measures ANOVA (by considering time as a repeated factor and stress as an independent factor) were performed as appropriate and followed by a Tukey post hoc test using GraphPad Prism program package (Version 6). P-value below 0.05 was considered as the level of significance.

## Supplementary Information

Supplementary Information 1.

Supplementary Information 2.
