# Supplementary material for: Maternal stress induced endoplasmic reticulum stress and impaired pancreatic islets’ insulin secretion via glucocorticoid receptor upregulation in adult male rat offspring
Source: Sci Rep. 2022 Jul 22;12:12552. doi: 10.1038/s41598-022-16621-5 (PMC9307850; doi:10.1038/s41598-022-16621-5)

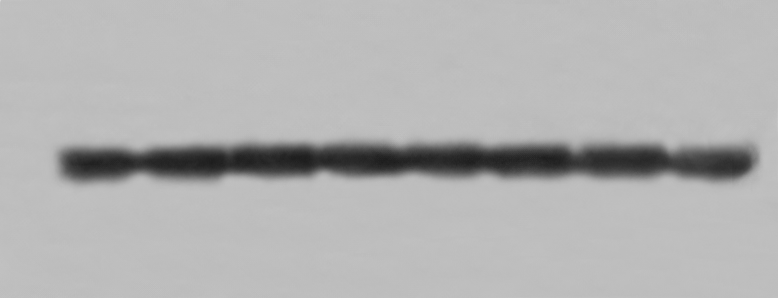
Original western blot of GR, and β-actin proteins are shown as follows:

**CTRL**

**35 KDa**

**45 KDa**

**PPLS**

**PLS**

**PPPS**

**LS**

**PS**

**PPS**

**PPPLS**

**β-actin**

**GR**

**125 KDa**

**PPLS**

**PLS**

**PPPS**

**PPS**

**PPPLS**

**CTRL**


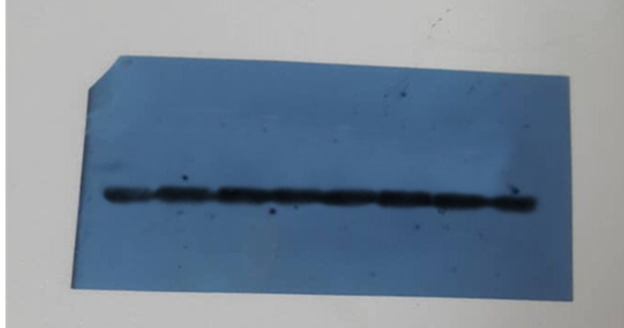

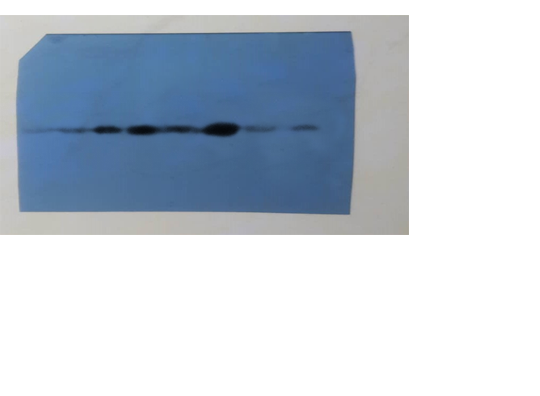

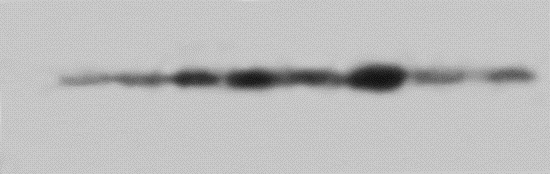


**GR**

**β-actin**

**PS**

**LS**

**90 KDa**

Original western blot of Bip, Chop, WFS1 and Calnexin proteins are shown as follows:


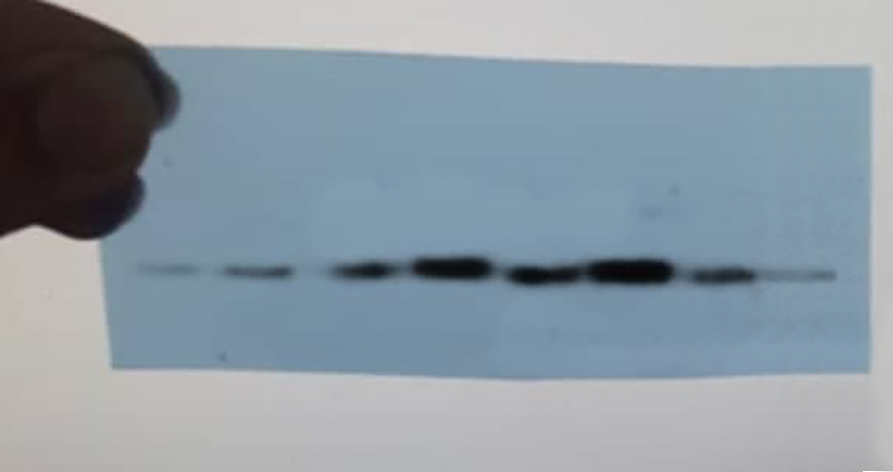

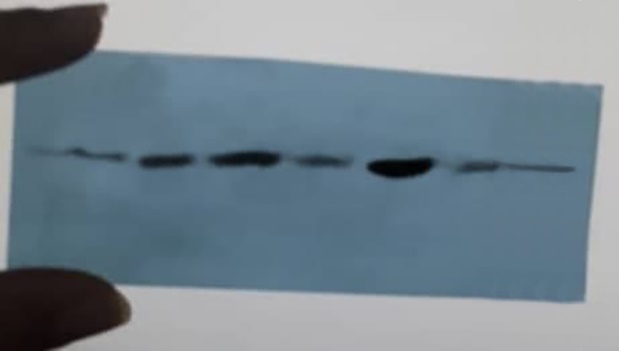

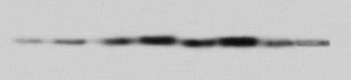

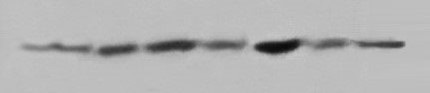


**Chop**

**Bip**

**PS**

**LS**

**PLS**

**PPPLS**

**PPLS**

**PPPS**

**PPS**

**CTRL**

**25 KDa**

**15 KDa**

**72 KDa**

**Chop**

**100 KDa**

**LS**

**Bip**

**PPLS**

**PLS**

**PPPS**

**PS**

**PPS**

**PPPLS**

**CTRL**


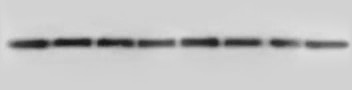


**Calnexin**

**72 KDa**

**100 KDa**

**PPLS**

**PLS**

**PPPS**

**LS**

**PS**

**PPS**

**PPPLS**

**CTRL**

**100 KDa**

**WFS1**


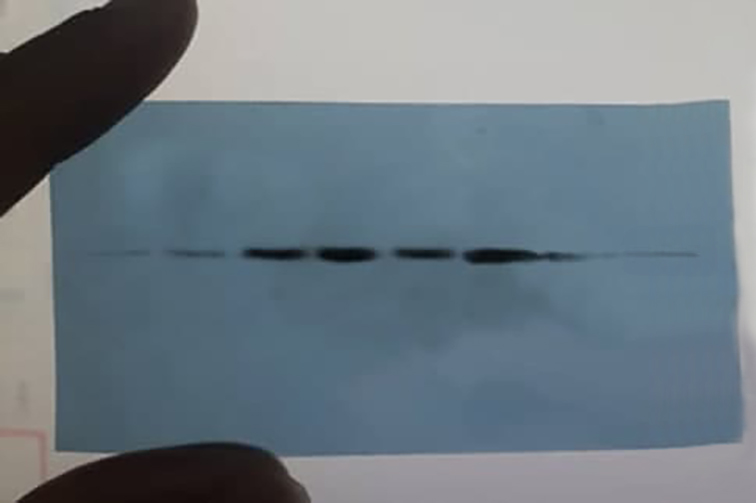


**WFS1**


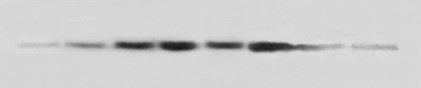


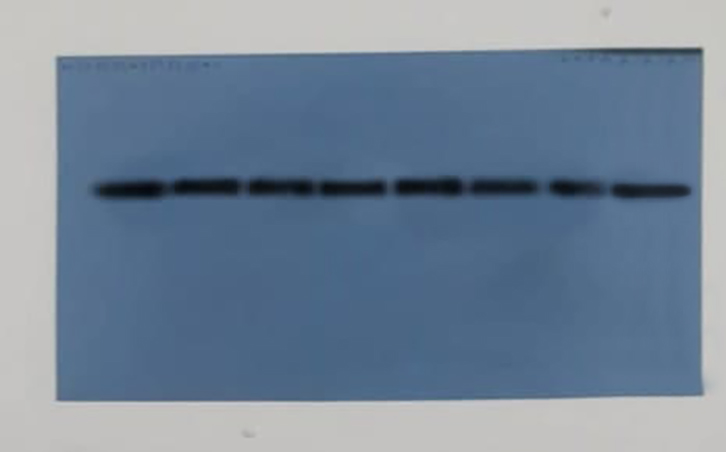


**Calnexin**

**PPLS**

**PLS**

**PPPS**

**LS**

**PS**

**PPS**

**PPPLS**

**CTRL**

Original agarose gel (2%) of PCR products from the DNA of WFS1 are shown as follows:


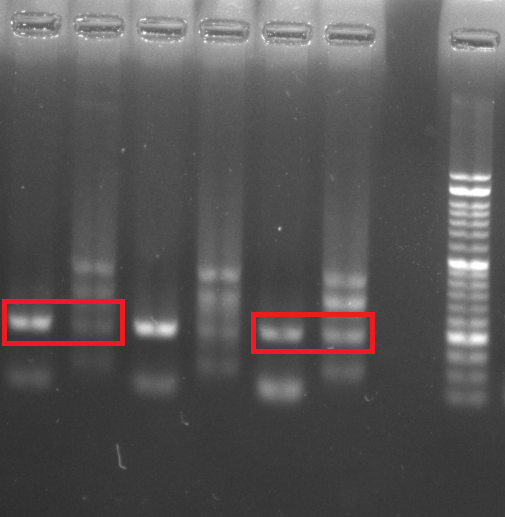

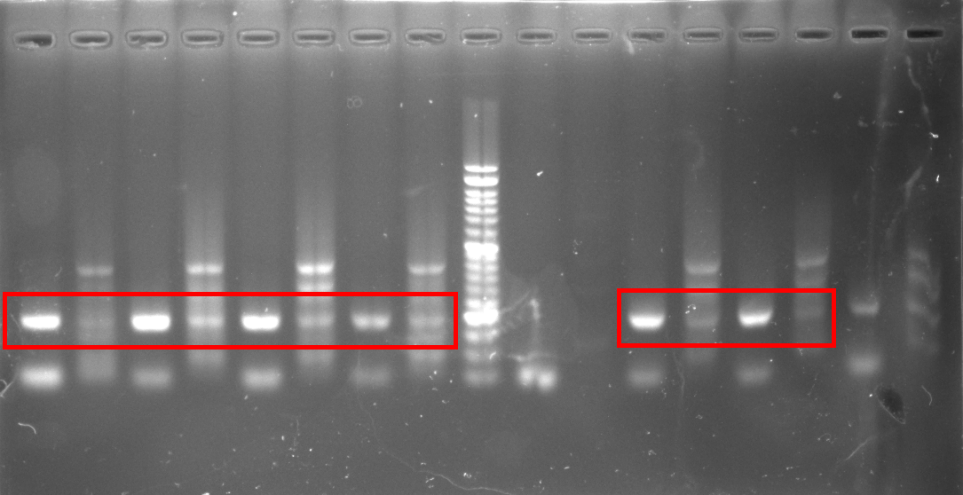


U

U

M

U

U

U

M

PPLS

M

LS

PS

M

PLS

PPPS

PPS

M

PPPLS

CTRL

M

M

**Marker 200 bp**

**Marker 200 bp**

**PPPLS**

**PS**

**U**

**M**

**WFS1**

U

M

U

U

**PPS**

**PLS**

**PPLS**

**LS**

**PPPS**

**CTRL**

**M**

**U**

**M**

**U**

**M**

**U**

**M**

**U**

**M**

**U**

**U**

**M**

**U**

**M**


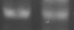

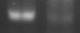

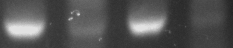

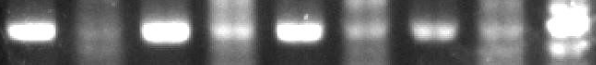

Supplement: Supplementary file 1 — Supplementary Information 1. [file 41598_2022_16621_MOESM1_ESM.docx]
