# Supplementary material for: Maternal stress induced endoplasmic reticulum stress and impaired pancreatic islets’ insulin secretion via glucocorticoid receptor upregulation in adult male rat offspring
Source: Sci Rep. 2022 Jul 22;12:12552. doi: 10.1038/s41598-022-16621-5 (PMC9307850; doi:10.1038/s41598-022-16621-5)
Supplement: Supplementary file 2 — Supplementary Information 2. [file 41598_2022_16621_MOESM2_ESM.docx]

**Supplementary Material and Methods**

**Islet isolation procedure**

The distal end of the common bile duct adjacent to the duodenum was clamped to occlude the bile pathway to the duodenum, the duct was cannulated with a polyethylene catheter (Portex Intravenous Cannula 2.5 F, 0.75 mm OD), and 10 ml cold Hank’s buffer (containing in mM: NaC1, 137; KCI, 5.4; CaCl_2_, 1.2; MgSO_4_7H_2_O, 0.8; Na_2_HPO_4_.2H_2_O, 0.3; KH_2_PO_4_, 0.4; NaHCO_3_, 4.2 (Merck, Germany)[^1^](#_ENREF_1) , containing 0.45 mg/ml of collagenase P (Roche, Cat. # 11213 865 001, Germany) was injected into the duct. The inflated pancreas was removed and placed into a Petri dish and cleaned from fat and connective tissue. Then the pancreas was placed into a 50 ml falcon tube and incubated in water bath at 37°C for 17 min. Digestion was stopped by the addition of 40 ml cold Hank’s solution. The tube was hand shaken for 1 min and the suspension was dispensed into a glass container (7.5 cm diameter and 4.5 cm height). Cold Hank’s solution was added and aspirated after precipitation. After three times washing with cold Hank’s solution, the islets were hand-picked under a stereomicroscope (Blue Light, USA). The islets were used freshly for studies related to insulin secretion [^2^](#_ENREF_2).

**Isolated islets’ Glucose-stimulated insulin secretion content**

From the isolated islets of each animal (n=4 rats/group), 2 groups of ten islets for each glucose concentration were picked (second-picking) and placed in the plastic cups (8 cups into total for each condition). All procedures for islet separation were carried out on the ice tray. After removing the excess Hank’s solution, 1 ml of Krebs-Ringer solution (pH = 7.4) [containing in mM: NaCl, 111; KCI, 5; MgCl_2_ 6H_2_O, 1; CaCl_2_, 1; NaHCO_3_, 24 (Merck, Germany); Hepes, 10 and BSA,0.5g/dl (Sigma, USA)] containing 5.6 or 16.7 mM glucose was added to the cups and incubated for 90 min in 37°C water bath while the cups were gassed with 95 % O2 and 5 % CO2 for 5 min at the beginning. Then the supernatant was removed and stored at –80°C to measure the secreted insulin. In order to assess the islets’ insulin content, 1 ml of acid-ethanol (0.18 M HCl in 70% ethanol) was added to the islets after removing the supernatant and kept overnight at 4°C. Finally, the supernatant was removed after centrifugation at 1300 ×g for 10 min and its insulin and total protein contents were assessed [^3^](#_ENREF_3).

**Measurement of protein levels in pancreatic rough endoplasmic reticulum (RER) and pancreatic tissue**

The pancreatic samples were homogenized in 2 ml of a lysis buffer containing complete protease inhibitor cocktail and centrifuged at 1250 ×g for 15 min to remove the cell debris. Moreover, rough vesicles derived from RER of rat pancreatic cells were extracted by the method as described by Kan et al [^4^](#_ENREF_4). The supernatant was collected to quantify its total protein concentration using the Bradford method [^5^](#_ENREF_5). The proteins were loaded and electrophoresed on polyacrylamide gels containing 12% sodium dodecyl sulfate (SDS) and then transferred to a PVDF (polyvinylidene fluoride) membrane. The membranes were incubated overnight (at 4 °C) with the primary antibody at 1:1000 dilution. The next day the membranes were incubated for 90 min at room temperature with the secondary antibody (Anti-rabbit IgG, Sc-516102) and were then visualized using ECL advance kit (Amershom Bioscience, USA). The quantification of the results was performed with a densitometry scan of the films, and data analysis was performed using Image J. The results were expressed as a relative ratio of the target protein to the reference protein. Calnexin is a marker of ER and were used as an internal loading control for the pancreatic extracted ER. β-actin was used as internal loading for the pancreatic tissue.

**Measurement of Bip, Chop, WFS1, GR mRNA Levels in pancreatic tissue**

Total RNA of the pancreas was extracted using the RiboEx RNA Purification Kit (GeneAll, Korea) according to manufacturer's instructions. The quantity and the purity of RNA samples were assessed using Nano drop spectrophotometer (Thermo Fisher Scientific, USA). Complementary DNA (cDNA) synthesis was performed with 1μg of total RNA sample using cDNA synthesis kit (YTA, IRAN) according to manufacturer’s protocol. For QRT-PCR, primers were designed using the Oligo7 software and QRT-PCR performed by ABI Step One Real-time PCR system (Applied Biosystems, USA). All reactions were set up in 13 µL volumes and contained 1 µL of cDNA, 0.3 µL of each primer, 7.5 µL of Master Mix SYBR Green high ROX (Ampliqon, Denmark), and 4.5 µL nuclease-free water. Amplification conditions were: 95 °C for 10 min (activation), followed by 40 cycles with 95 °C for 20s (denaturation), 58 °C for 1 min, 95 °C for 15s (annealing) and 60 °C for 1min (extension). The average cycle threshold (Ct) was determined for each sample and normalized to beta-actin. The relative fold change was calculated by the (2^ (-ΔΔCT)) method.

**Reference**

1. Zardooz, H., Asl, S.Z. & Naseri, M.G. Effect of chronic psychological stress on insulin release from rat isolated pancreatic islets. *Life Sci.* **79**, 57-62 (2006).

2. Sadeghimahalli, F., Karbaschi, R., Zardooz, H., Khodagholi, F. & Rostamkhani, F. Effect of early life stress on pancreatic isolated islets’ insulin secretion in young adult male rats subjected to chronic stress. *Endocrine* **48**, 493-503 (2015).

3. Darnaudéry, M., Dutriez, I., Viltart, O., Morley-Fletcher, S. & Maccari, S. Stress during gestation induces lasting effects on emotional reactivity of the dam rat. *Behav. Brain Res.* **153**, 211-216 (2004).

4. Kan, F.W., Jolicoeur, M. & Paiement, J. Freeze-fracture analysis of the effects of intermediates of the phosphatidylinositol cycle on fusion of rough endoplasmic reticulum membranes. *Biochim. Biophys. Acta - Biomembr.* **1107**, 331-341 (1992).

5. Kruger, N.J. The Bradford method for protein quantitation. *The protein protocols handbook*, 17-24 (2009).
